# Supplementary material for: Conservation of the cooling agent binding pocket within the TRPM subfamily
Source: bioRxiv. 2024 Aug 21:2024.05.20.595003. Originally published 2024 May 21. Preprint. [Version 2] doi: 10.1101/2024.05.20.595003 (PMC11142142; doi:10.1101/2024.05.20.595003)
Supplement: Supplement 1 [file NIHPP2024.05.20.595003v2-supplement-1.pdf]

bioRxiv preprint doi: https://doi.org/10.1101/2024.05.20.595003; this version posted August 21, 2024. The copyright holder for this preprint (which was not certified by peer review) is the author/funder. This article is a US Government work. It is not subject to copyright under 17 USC 105 and is also made available for use under a CC0 license.

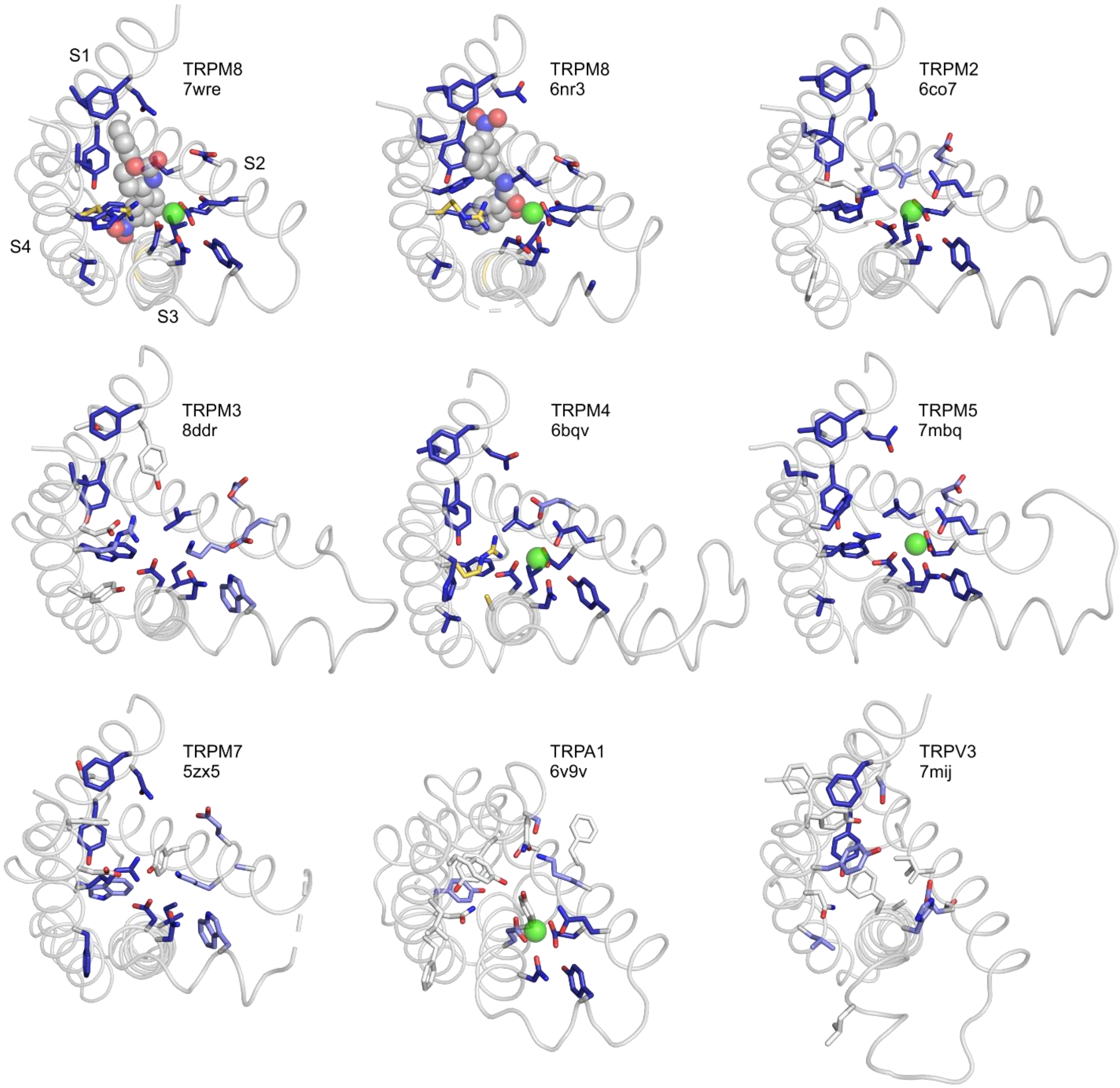

Figure 2 – Figure Supplement 1

S1-S4 residues contributing to the icilin binding pocket in TRPM8 structures 7wre and 6nr3 are shown as blue licorice, viewed from the intracellular side of the membrane, with the TRP box omitted for clarity. Cooling agent binding pocket mutations used in the present study are shown with carbon atoms colored gold and labeled in TRPM8 and TRPM4, and the equivalent residues in other channels are colored based on the alignment quality score, as in Figure 2A.

bioRxiv preprint doi: https://doi.org/10.1101/2024.05.20.595003; this version posted August 21, 2024. The copyright holder for this preprint (which was not certified by peer review) is the author/funder. This article is a US Government work. It is not subject to copyright under 17 USC 105 and is also made available for use under a CC0 license.

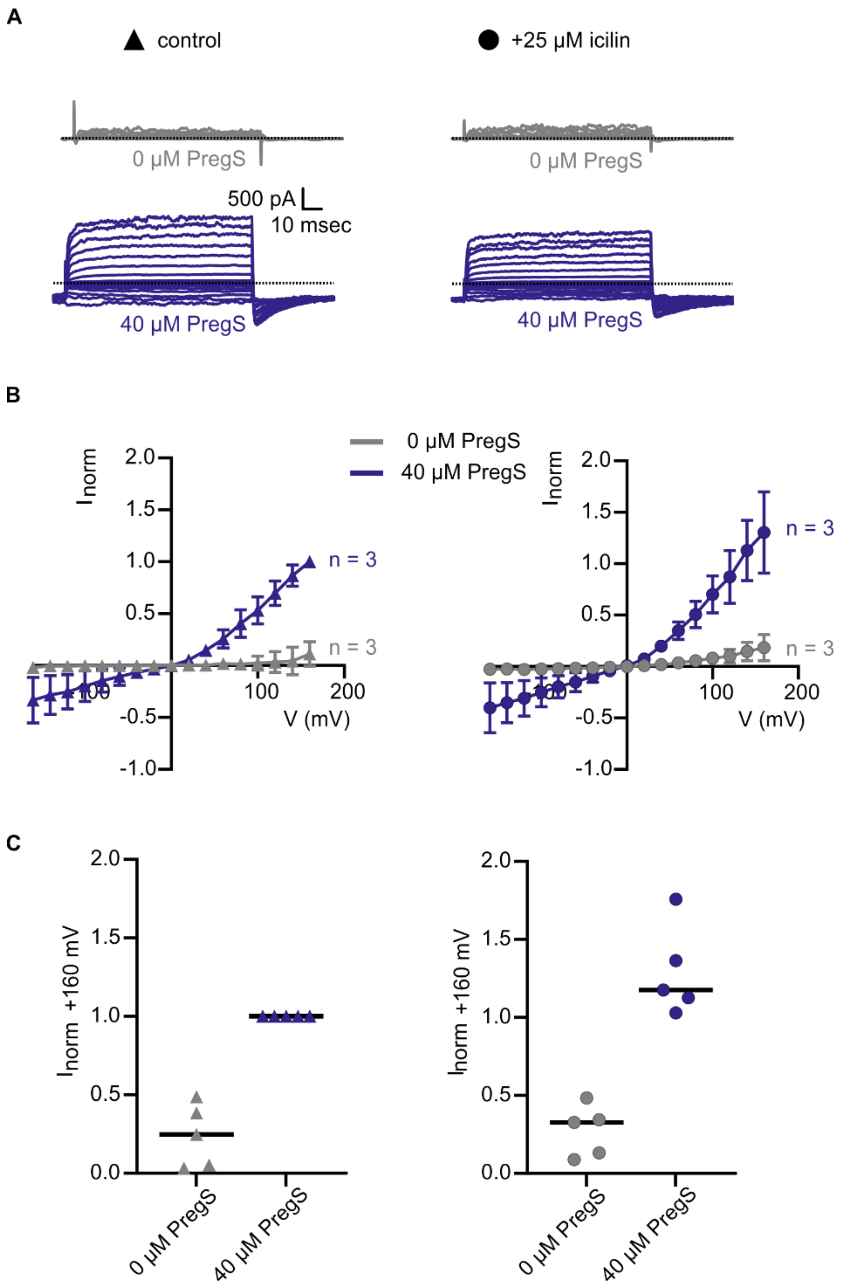

bioRxiv preprint doi: https://doi.org/10.1101/2024.05.20.595003; this version posted August 21, 2024. The copyright holder for this preprint (which was not certified by peer review) is the author/funder. This article is a US Government work. It is not subject to copyright under 17 USC 105 and is also made available for use under a CC0 license.

← Figure 4 – Figure Supplement 1 | Icilin does not modulate voltage-dependent activation of TRPM3α2.

A) Sample current families obtained using a holding voltage of -60 mV with 50 msec prepulses to -150 mV before 100 msec steps to voltages between -160 mV and +160 mV (Δ 20 mV), and then returning to -60 mV. Control traces in the left column were obtained with TRPM3α2 in the absence of icilin (left, triangles), and traces in the right column were obtained in the presence of 25 µM icilin (right, circles). Activation of TRPM3 current is achieved with 40 µM pregnenolone sulphate (PregS, navy). **B)** Corresponding normalized I-V relations and **C)** plots of normalized current evoked at +160 mV in populations of cells in the absence (left, triangles) or presence (right, circles) of 25 µM icilin. Absolute values were obtained from steady-state current measurements. For each cell, values are normalized to the absolute current at +160 mV in the presence of 40 µM PregS. Error bars indicate standard error of the mean.
