# Supplementary material for: Conservation of the cooling agent binding pocket within the TRPM subfamily
Source: bioRxiv. 2024 May 21:2024.05.20.595003. Preprint. [Version 1] doi: 10.1101/2024.05.20.595003 (PMC11142142; doi:10.1101/2024.05.20.595003)
Supplement: Supplementary file 1 [file NIHPP2024.05.20.595003v1-supplement-1.pdf]

bioRxiv preprint doi: https://doi.org/10.1101/2024.05.20.595003; this version posted May 21, 2024. The copyright holder for this preprint (which was not certified by peer review) is the author/funder. This article is a US Government work. It is not subject to copyright under 17 USC 105 and is also made available for use under a CC0 license.

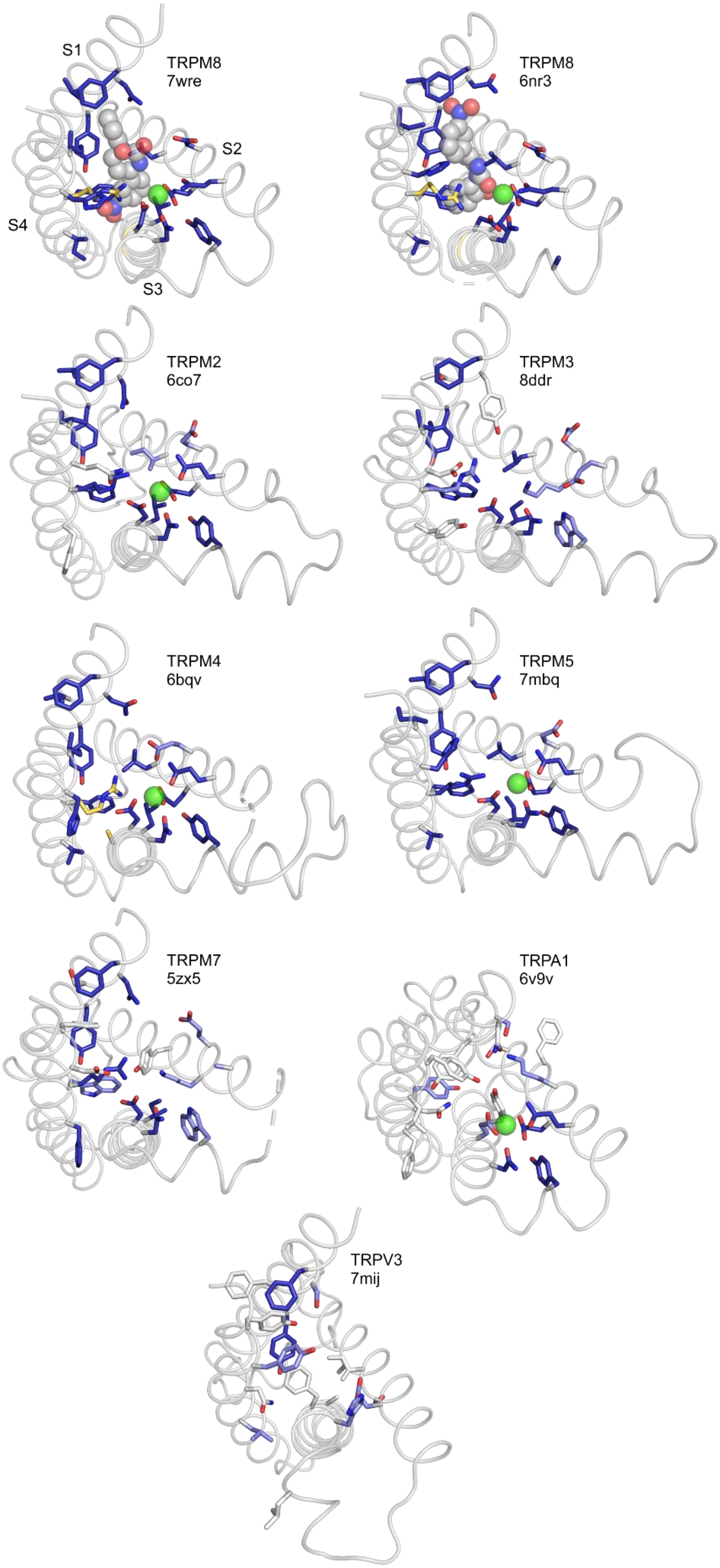

bioRxiv preprint doi: https://doi.org/10.1101/2024.05.20.595003; this version posted May 21, 2024. The copyright holder for this preprint (which was not certified by peer review) is the author/funder. This article is a US Government work. It is not subject to copyright under 17 USC 105 and is also made available for use under a CC0 license.

← Figure 2 – Figure Supplement 1

S1-S4 residues contributing to the icilin binding pocket in TRPM8 structures 7wre and 6nr3 are shown as blue licorice, viewed from the intracellular side of the membrane, with the TRP box omitted for clarity. Cooling agent binding pocket mutations used in the present study are shown with carbon atoms colored gold and labeled in TRPM8 and TRPM4, and the equivalent residues in other channels are colored based on the alignment quality score, as in Figure 2A.
